# Interacting Effects of Insects and Flooding on Wood Decomposition

**DOI:** 10.1371/journal.pone.0101867

**Published:** 2014-07-10

**Authors:** Michael D. Ulyshen

**Affiliations:** Southern Research Station, United States Department of Agriculture, Forest Service, Starkville, Mississippi, United States of America; Roehampton university, United Kingdom

## Abstract

Saproxylic arthropods are thought to play an important role in wood decomposition but very few efforts have been made to quantify their contributions to the process and the factors controlling their activities are not well understood. In the current study, mesh exclusion bags were used to quantify how arthropods affect loblolly pine (*Pinus taeda* L.) decomposition rates in both seasonally flooded and unflooded forests over a 31-month period in the southeastern United States. Wood specific gravity (based on initial wood volume) was significantly lower in bolts placed in unflooded forests and for those unprotected from insects. Approximately 20.5% and 13.7% of specific gravity loss after 31 months was attributable to insect activity in flooded and unflooded forests, respectively. Importantly, minimal between-treatment differences in water content and the results from a novel test carried out separately suggest the mesh bags had no significant impact on wood mass loss beyond the exclusion of insects. Subterranean termites (Isoptera: Rhinotermitidae: *Reticulitermes* spp.) were 5–6 times more active below-ground in unflooded forests compared to flooded forests based on wooden monitoring stakes. They were also slightly more active above-ground in unflooded forests but these differences were not statistically significant. Similarly, seasonal flooding had no detectable effect on above-ground beetle (Coleoptera) richness or abundance. Although seasonal flooding strongly reduced *Reticulitermes* activity below-ground, it can be concluded from an insignificant interaction between forest type and exclusion treatment that reduced above-ground decomposition rates in seasonally flooded forests were due largely to suppressed microbial activity at those locations. The findings from this study indicate that southeastern U.S. arthropod communities accelerate above-ground wood decomposition significantly and to a similar extent in both flooded and unflooded forests. Seasonal flooding has the potential to substantially reduce the contributions of these organisms to wood decomposition below-ground, however.

## Introduction

Dead wood accounts for approximately 10–25% of above-ground forest biomass [1], [2] and provides habitat and resources for an equally or even larger proportion of forest biodiversity [3]. Factors affecting the size of the dead wood pool are therefore of great interest, especially with respect to carbon budgets and climate change [4]. Wood decomposition rates are thought to be determined primarily by microbial activity which, in turn, is governed by climatic factors, soil properties and resource quality [5], [6]. A wide variety of arthropod taxa are also known to consume or otherwise excavate dead wood and some of these–most notably termites [7]–[10] and wood-boring beetles [11]–[13]–are thought to significantly accelerate decomposition. Efforts to quantify this effect are few, however, and it remains entirely unknown how this influence may vary with physical conditions. Such research is needed to refine decomposition models which have generally ignored arthropod effects [14], [15] and would help to properly recognize the ecosystem services provided by this diverse and vulnerable fauna [16].

Moisture is perhaps the most important physical determinant of wood decomposition. Decomposition practically ceases under highly desiccating conditions, for example, as evidenced by millennia-old wood at high altitudes [17]. The same is true at the other extreme of the moisture gradient, as illustrated by very slow rates of decomposition in submerged [18] or saturated [19] wood. More moderate differences in wood moisture are known to be influential as well, e.g., wood appears to decompose more slowly in seasonally flooded forests than unflooded forests [20], [21], presumably due to the negative effects of periodic inundation on microbial communities [22]–[24]. Flooding has also been shown to negatively affect termites [25]–[28] and other wood-dwelling arthropods [29], [30] and this, in turn, has the potential to further influence decomposition rates.

Due to an abundance of both seasonally flooded and unflooded forests, the southeastern United States is an ideal location to test how flooding and arthropods interact to influence wood decomposition. This region supports a diverse assemblage of wood-dwelling beetles and several species of subterranean termites (*Reticulitermes* spp.) [31]. Both taxa appear to be influenced by seasonal flooding, with compositional differences in beetle and termite communities between flooded and unflooded forests [32], [33] and evidence that termite incidence is reduced at flooded sites [20], [34]. Only three previous efforts have been made to quantify the contributions of arthropods to wood decomposition in southeastern U.S. forests. Whereas two studies using wooden blocks found termites to have a significant effect on mass loss [9], [35], a five year exclusion study using pine bolts detected no such effect even though insects (primarily termites) consumed 15–20% of the wood volume in unprotected bolts [36]. Because the exclusion methods used in the latter study–a combination of insecticide treatment and mesh cages–may have affected wood decomposition (e.g., microbial activity) beyond the exclusion of insects, the findings for mass loss are inconclusive. This is a common problem among exclusion studies, prompting Kampichler and Bruckner [37] to call for additional experimentation to test for such unintended effects.

The current study sought to quantify the contributions of arthropods to wood decomposition in both seasonally flooded and unflooded forests using mesh bags designed to exclude insects. The incidence of termites was compared between forest types to determine the extent to which flooding reduced *Reticulitermes* activity. I hypothesized that 1) termites would be significantly less active in flooded forests, 2) insect exclusion would significantly reduce decomposition rates and 3) insect exclusion would have less of an effect on decomposition rates in flooded forests than in unflooded forests, i.e., a significant interaction between exclusion treatment and forest type. Finally, in response to Kampichler and Bruckner [37], I tested a novel method aimed at determining whether the mesh bags used to exclude insects in this study had any unintended effects on wood decomposition.

## Methods

### Location and design

**Figure 1:**
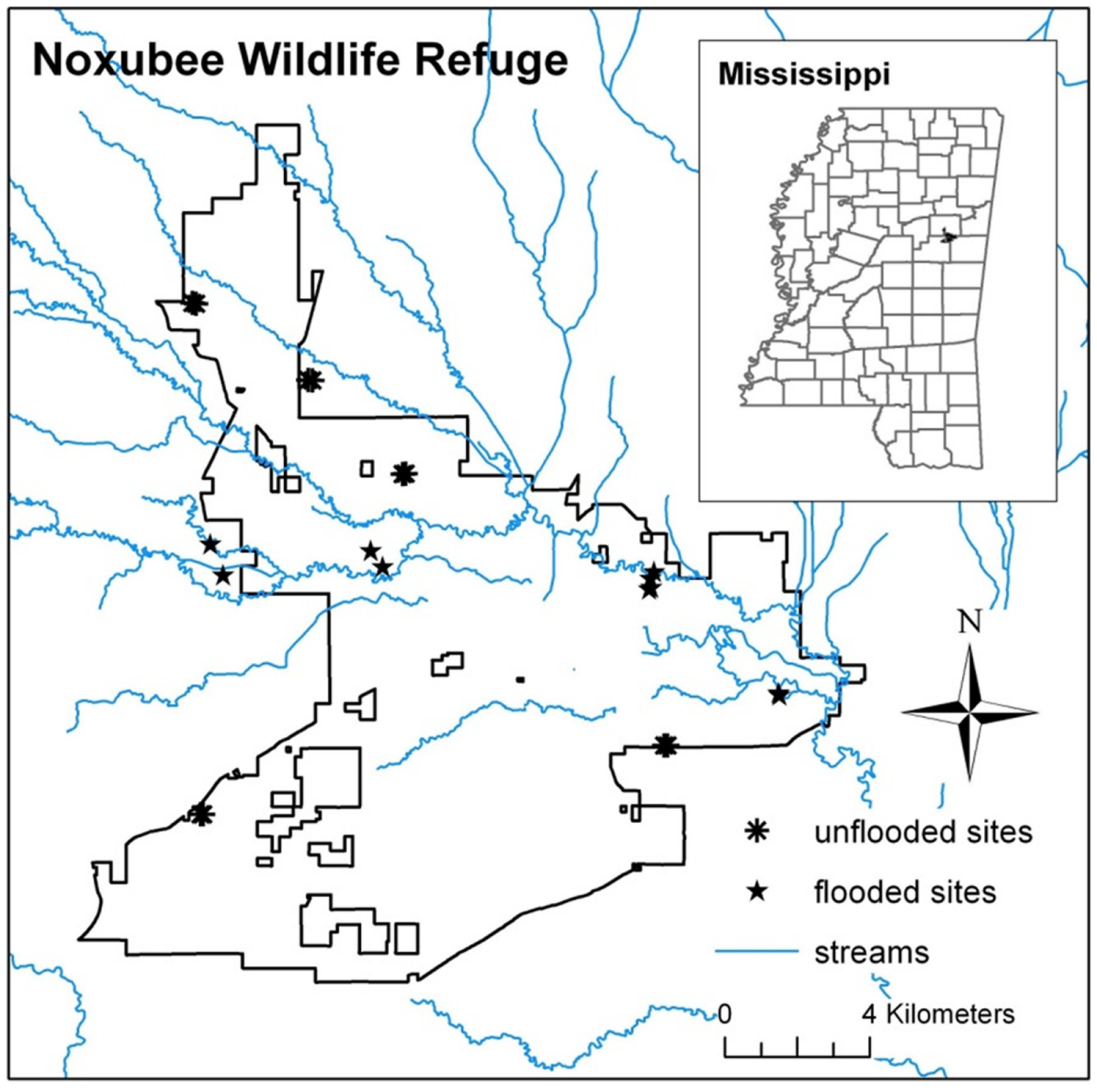
Map of study locations in northeastern Mississippi (Note: some locations are too close together to appear individually in this figure).

**Figure 2:**
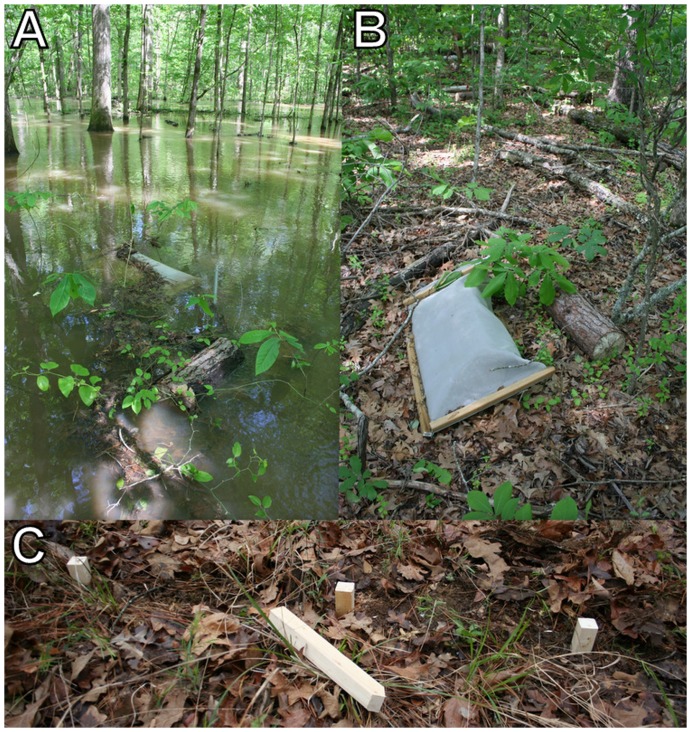
A pair of protected and unprotected bolts at a flooded (A) and unflooded (B) location in northeastern Mississippi, U.S.A. Wooden stakes were used to compare *Reticulitermes* activity between forest types (C).

Twenty locations dominated by mature mixed hardwood/pine forests were selected on the Noxubee National Wildlife Refuge (permission granted by Henry Sansing) and John W. Starr Memorial Forest (permission granted by Misty Booth) in Oktibbeha, Noxubee and Winston counties, Mississippi, U.S.A. Half of these were situated near streams and flooded from late winter to early spring every year. The other locations were situated at slightly higher elevations and never flooded. The locations were widely separated (100 m to 20 km) across the study area (Figure 1). A linear transect was established at each location beginning at least 20 m from the forest edge and running perpendicular to the edge. Each transect consisted of five plots separated by 10 m. The 200 bolts (i.e., experimental units) used in the study, measuring 55.9 cm long (i.e., 22 inches) and 23.1±0.2 cm in diameter, were cut from twenty loblolly pine trees felled locally in November 2010. The trees were ∼20 years old based on growth rings and lacked heartwood. One 5 cm-thick disk was removed from the base of each tree for initial specific gravity measurements. Each bolt was randomly assigned to a particular forest type, transect, plot and treatment. The treatments included bolts that were or were not enclosed within mesh bags, hereafter referred to as “protected” and “unprotected”, respectively. To construct the mesh bags, a pneumatic stapler loaded with 2.5 cm galvanized staples was used to securely fasten the stainless steel mesh (0.38 mm openings) to lengths of treated wood. The completed mesh bags measured approximately 119.4×64.8 cm. After a bolt was placed in a mesh bag, the screen at the open end was folded and stapled to a third piece of wood to achieve a complete seal. In early November 2010, each plot within each transect received one protected and one unprotected bolt (Figure 2A,B). Those placed in the flooded forests were secured to a metal stake driven into the ground using metal wire and eye screws. One pair of bolts was sampled about every six months from each transect beginning and ending at 6 and 31 months, respectively, for a total of five sampling periods. The first pair of bolts was taken from the first location in each transect (i.e., closest to the forest edge) and subsequent samples were taken sequentially along each transect. This sampling design was adopted to reduce visibility after one of the mesh bags was vandalized soon after the study began. Because the transects began at least 20 m from the edge and previous research has shown that proximity to the forest edge (e.g., 5 vs 55 m) has no influence on wood decomposition [38], edge effects were probably not an issue in the current study.

### Wood measurements

Upon collection, two ∼3.81 cm (i.e., 1.5 inch) disks were cut from one end of each bolt. The inner disk was used in wood measurements. Wood water content and density calculations were made using the following protocol: 1) each disk was debarked and then weighed upon collection to determine its wet weight; 2) the “initial” external volume of each disk was determined before drying by measuring its average thickness with calipers and the surface area of one face (note: Ulyshen et al. [36] showed that cross-sectional area does not change within the first 44 months of loblolly pine decomposition) by weighing cut-out tracings made on paper of known density; 3) each disk was dried for 24 hrs at 102°C and weighed immediately thereafter to determine its final dry weight; 4) each disk was submerged in a pan of water placed on a scale for one hour (allowing any trapped air to be displaced by water) to determine the final wood volume (i.e., ignoring wood consumed by insects, see below); and 5) disks exhibiting heavy *Reticulitermes* damage were burned completely to isolate soil carried into the wood by these insects [39]. Disk weights were corrected by subtracting dried soil weights. Water content was calculated on a dry weight basis (i.e., (wet weight- dry weight)/dry weight).

Two separate specific gravity measurements were made, depending on whether the initial or final wood volume was used in the denominator. The former calculation, hereafter referred to as “specific gravity (initial volume)” relates wood mass remaining to initial wood volume, including any wood missing due to invertebrate activity [39]. The latter calculation, hereafter referred to as “specific gravity (final volume)”, quantifies the specific gravity of the remaining wood (i.e., the wood not consumed by invertebrates). Specific gravity (initial volume) was calculated from the dry wood weight (without soil) and the external disk volume measured as described above. Specific gravity (final volume) was calculated using the water-displaced volume (see above). To correct for the influence of *Reticulitermes*-imported soil, soil volume (i.e., soil dry weight/soil density) was subtracted from the water-displaced volume prior to making these calculations.

### *Reticulitermes* and beetle activity

Three methods were used to compare *Reticulitermes* activity between flooded and unflooded forests. First, subterranean activity was compared by driving three sharpened spruce stakes (3.8×3.8×30.5 cm) 22.9 cm into the ground half-way between each pair of plots in each transect (i.e., a total of 12 stakes at each of the 20 locations). The three stakes within each group were separated by 0.5 m (Figure 2C). One stake from each group was collected 3, 6 and 12 months after installation in April 2011 (i.e., beginning about six months after the experimental bolts were distributed) and scored for the presence or absence of feeding damage. I also compared *Reticulitermes* activity in the experimental bolts by recording the presence or absence of visible *Reticulitermes* damage on the disks for each sampling period. In addition, a comparison between forest types was based on whether or not termites emerged from the bolts placed in rearing bags (see below). The species richness and abundance of beetles emerging from unprotected bolts after 6 months were compared between forest types.

### Mesh bag effects

To determine how effective the mesh bags were at excluding arthropods, beetles and termites were sampled from both protected and unprotected bolts after six months in the field. After collecting the disks from the ends of the bolts (for the wood measurements, see above), the remaining bolt sections were suspended in rearing bags [32] to collect emerging insects over a six-month period. All collected beetles were identified to morphospecies and counted. The presence or absence of termites was also recorded. Additional data on the presence or absence of termites were collected after 18 months using the same methods.

**Figure 3:**
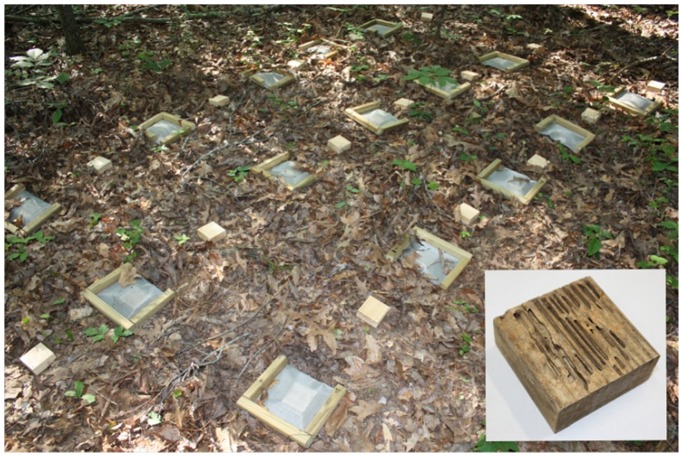
Protected and unprotected blocks without termite damage were used to determine how the mesh bags affected water content beyond the exclusion of insects. An unprotected block with *Reticulitermes* damage is shown in the inset.

In addition, a separate field study was conducted to determine how the mesh bags may have affected wood water content (dry weight basis) and mass loss rates beyond the exclusion of insects. The same materials were used to construct small mesh bags (9×11 inches) large enough to receive one 8.8×8.8×3.5 cm block of untreated pine. Unprotected blocks were used as a reference and all blocks were weighed at the beginning of the experiment, after drying them for 24 hrs at 102 C. Fifteen replicates of each treatment were placed in contact with the soil at a single forested location for 20 weeks (April 26–September 13, 2012). The protected and unprotected blocks were placed in a grid in a single forest, separated by 0.5 m and arranged in an alternating fashion (Fig. 3). The unprotected blocks were examined carefully at the time of collection and categorized based on whether they had been attacked by arthropods (termites) or not. To isolate the effect of the mesh bags from those of arthropods, comparisons were made between protected blocks and unprotected blocks that had no visible evidence of arthropod feeding. Wooden blocks were used instead of a more natural substrate in order to more easily inspect for arthropod damage.

### Statistical analysis

Data from two of the 200 original bolts were excluded from the final dataset due damage to the mesh bags (vandalism and a fallen tree limb). Analyses of variance were carried out on the remaining data using the proc mixed procedure of SAS [40] to determine how forest type (flooded vs. unflooded), treatment (protected vs. unprotected) and time (6, 12, 18, 24, and 31 months) affected specific gravity (initial volume and final volume) and water content. Random effects included transect(forest), treatment*transect(forest) and time*transect(forest) and denominator degrees of freedom were obtained using the Kenward-Roger method [40]. Initial bolt diameter was included as a covariate in all models. Water content and initial bolt diameter were log-transformed to satisfy normality assumptions. In addition, effect sizes (Hedges’ d) and their 95% confidence intervals [41]–[43] were calculated to compare specific gravity (initial volume), specific gravity (final volume) and water content between treatments (i.e., protected-unprotected) for each combination of forest type and sample period. Effect sizes were considered non-significant when their confidence intervals overlapped zero.

For the comparisons of *Reticulitermes* activity between forest types, the Chi Square test was used on presence/absence data from the wooden stakes and Fisher’s exact test was used on presence/absence data from the wooden disks. In addition, ANOVA was used to compare the species richness and log-transformed abundance of beetles emerging from unprotected bolts after six months between forest types.

Finally, the Kruskal-Wallis Test was used to compare wood water content and mass loss among the three types of blocks (i.e., protected, unprotected with *Reticulitermes* attack and unprotected without *Reticulitermes* attack) from the separate mesh bag study.

## Results

### Specific gravity

**Figure 4:**
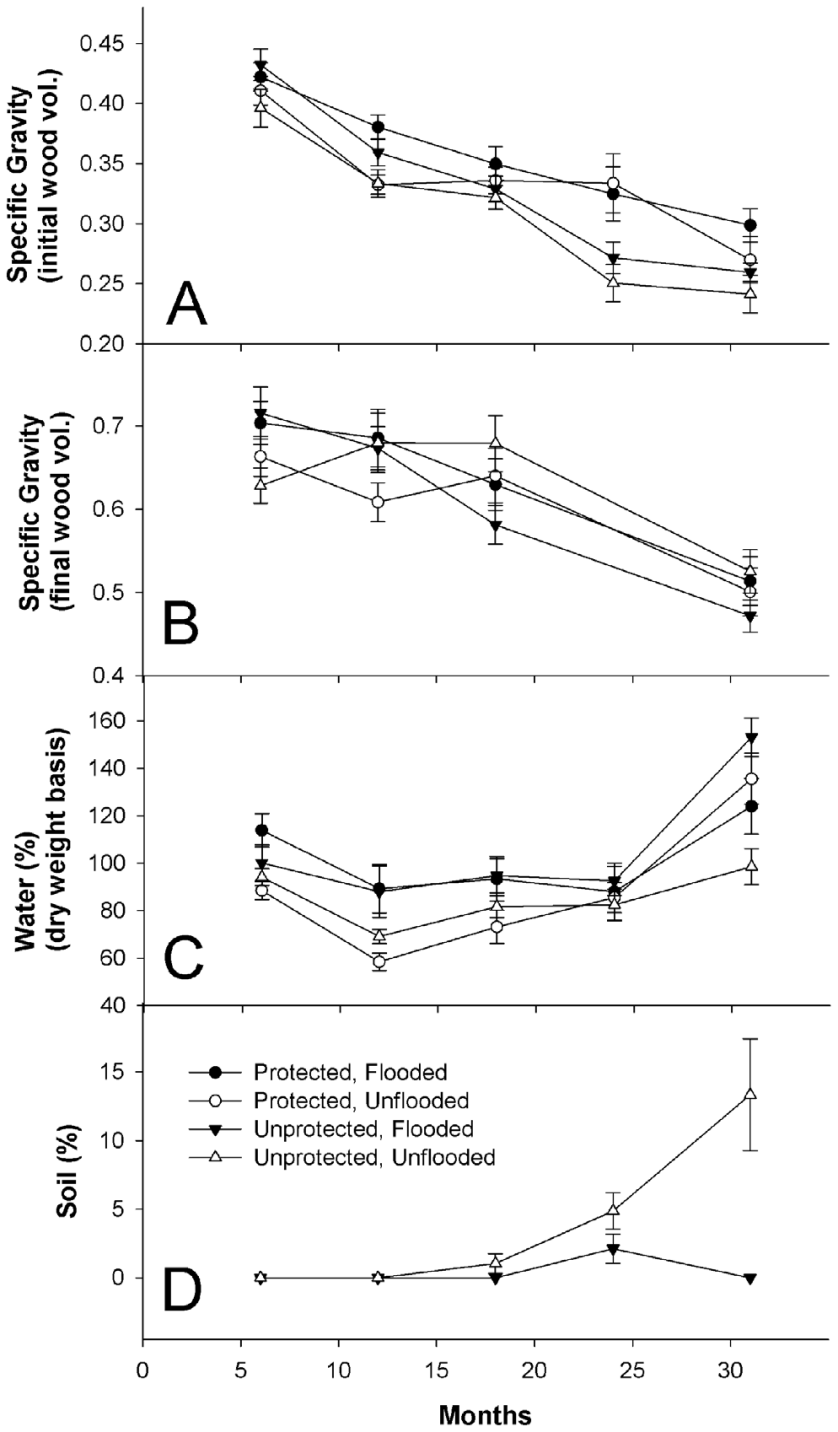
Mean ± SE specific gravity (initial wood volume) (A), specific gravity (final wood volume) (B), water content (C) and soil content (D). Note: data on specific gravity (final wood volume) were not collected at 24 months.

**Figure 5:**
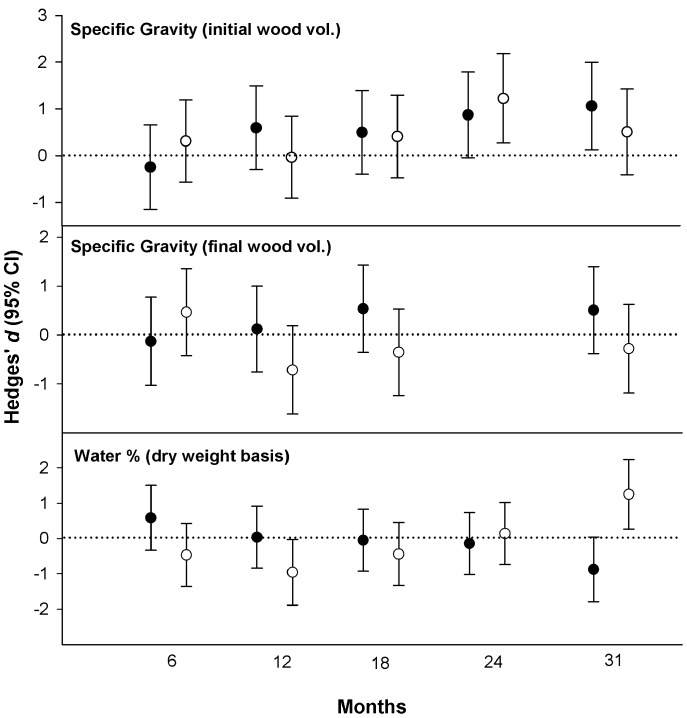
Effect sizes (Hedges’ d ±95% confidence intervals) for comparisons between protected and unprotected bolts (i.e., protected-unprotected) in flooded (closed circles) and unflooded (open circles) forests. Note: data on specific gravity (final wood volume) were not collected at 24 months.

Specific gravity (initial volume) varied significantly between forest types and treatments (Table 1), being generally lower in bolts placed in unflooded forests and for those unprotected from insects (Figure 4A and see Table 2 for details on decomposition rates). There was a significant treatment*time interaction due to specific gravity (initial volume) decreasing less steeply over time in protected bolts compared to unprotected bolts. Based on the 95% confidence intervals for effect size, specific gravity (initial volume) was significantly lower due to insects in unflooded forests at 24 months and in flooded forests at 31 months (Figure 5). At the end of the study, after 31 months, approximately 20.5% and 13.7% of specific gravity (initial volume) loss was attributable to arthropod activity [i.e., (SG_protected_−SG_unprotected_)/(SG_initial_−SC_unprotected_), with SG_initial_ = 0.45] in flooded and unflooded forests, respectively.

**Table 1:** Comparisons of response variables between forest types (flooded vs. unflooded), treatments (protected vs. unprotected) and over time (6, 12, 18, 24, and 31 months).

	specific gravity (initial volume)	specific gravity (final volume)	water (%) (dry wt. basis)
forest	**F_1,18.1_ = 7.44, p = 0.01**	F_1,17.8_ = 0.14, p = 0.71	**F_1,17.9_ = 6.37, p = 0.02**
treatment	**F_1,18_ = 17.38, p<0.001**	F_1,71.5_ = 0.0, p = 0.99	F_1,86.3_ = 0.48, p = 0.49
treatment*forest	F_1,18.1_ = 0.27, p = 0.61	F_1,72.3_ = 2.65, p = 0.11	F_1,86.4_ = 0.41, p = 0.52
time	**F_4,72_ = 71.81, p<0.0001**	**F_3,54.2_ = 30.65, p<0.0001**	**F_4,70_ = 26.21, p<0.0001**
forest*time	F_4,72.1_ = 0.85, p = 0.50	**F_3,54.3_ = 3.28, p = 0.03**	F_4,70_ = 1.12, p = 0.36
treatment*time	**F_4,72_ = 3.54, p = 0.01**	F_3,71.5_ = 0.6, p = 0.62	F_4,86.4_ = 0.96, p = 0.44
treatment*forest*time	F_4,72.2_ = 0.65, p = 0.63	F_3,71.6_ = 1.64, p = 0.19	**F_4,86.6_ = 6.71, p<0.0001**
initial bolt diameter	**F_1,171_ = 18.02, p<0.0001**	**F_1,127_ = 5.13, p = 0.03**	**F_1,154_ = 7.82, p<0.01**

Initial bolt diameter was included as a covariate. Significant results are shown in bold.

**Table 2:** Estimated decay rates and half-lives based on the single exponential decay model [3] and a mean initial specific gravity (based on samples collected when the trees were felled) of 0.45.

Forest	treatment	decay rate constant (months/years)	half life (months/years)
flooded	protected	0.013/0.157	53.0/4.4
flooded	unprotected	0.018/0.212	39.2/3.3
unflooded	protected	0.016/0.198	42.1/3.5
unflooded	unprotected	0.020/0.243	34.2/2.8

Specific gravity (final volume) did not vary significantly between forest types or treatments but did decrease significantly over time and there was a significant forest*time interaction (Table 1, Figure 4B). Based on the 95% confidence intervals for effect size, specific gravity (final volume) never varied significantly between treatments in either forest type (Figure 5).

### Water content

Water content varied significantly between forest types (Table 1), being higher in flooded forests (Figure 4C). Although there were no significant differences between treatments when averaged over forests and time, there was a significant treatment*forest*time interaction (Table 1). Water content also varied significantly over time. It remained relatively stable for the first four sampling periods before increasing sharply after 24 months (Figure 4C). Water content varied significantly between treatments in both forest types at 31 months, being significantly lower in protected bolts in flooded forests and significantly higher in protected bolts in unflooded forests (Figure 5).

### Soil content

Among unprotected bolts, *Reticulitermes*-imported soil content increased steadily after the first year in unflooded forests until the end of the study when it accounted for 13.3% of the dry wood weight (Figure 4D). By contrast, substantially less soil was recovered from unprotected bolts placed in flooded forests (Figure 4D).

### *Reticulitermes* and beetle activity

The percentage of wooden stakes attacked below-ground by termites was about 5–6 times greater in unflooded forests compared to flooded forests and the difference was significant for all three time periods (Table 3). Although *Reticulitermes* presence and activity in unprotected bolts were generally higher above-ground in unflooded forests as well, these differences were not significant (Table 3). Finally, much less *Reticulitermes*-imported soil was recovered from unprotected bolts in flooded forests compared to their unflooded counterparts (Figure 4D).

**Table 3:** Comparisons of *Reticulitermes* activity in wooden stakes and unprotected bolts between flooded and unflooded forests.

Measurement	Months	Flooded	Unflooded	Result
% wooden stakes attacked	3	7.5	45.0	χ^2^ (1, *N* = 80) = 14.5, *p*<0.0001
	6	10	52.5	χ^2^(1, *N* = 80) = 16.8, *p*<0.0001
	12	13.5	62.5	χ^2^(1, *N* = 75) = 21.3, *p*<0.0001
% disks with visible *Reticulitermes* damage	6	0	0	–
	12	50	90	*p* = 0.14
	18	30	60	*p* = 0.37
	24	70	100	*p* = 0.21
	31	70	100	*p* = 0.21
% bolts from which termites emerged	6	50	50	*p* = 1.0
	18	40	60	*p* = 0.66

Results for stakes were analyzed using a Chi Square test whereas those for disks and bolts were analyzed using Fisher’s exact test.

The mean ± SE number of beetle species to emerge from unprotected bolts after six months in the field was 10.2±0.7 and 10.4±1.1 for flooded and unflooded forests, respectively, and this was not statistically significant (F_1,18_ = 0.02, P = 0.88). Similarly, 373.1±148.2 and 136.1±38.3 individuals emerged from unprotected bolts from flooded and unflooded forests, respectively, and this difference was also not significant (F_1,18_ = 3.06, P = 0.10, ANOVA on log-transformed abundance data).

### Mesh bag effect

Based on insects emerging from bolts placed in rearing bags, 50% of the unprotected bolts in both flooded and unflooded forests had termites after six months whereas no termites were present in the protected bolts at that time. Similarly, after 18 months, 40% and 60% of unprotected bolts in flooded and unflooded forests, respectively, had termites whereas no termites were present in the protected bolts. In addition, large numbers of bark, ambrosia and wood-boring beetles emerged from the unprotected bolts after six months but none emerged from protected bolts. Not all beetles were excluded by the mesh bags, however. Of the 5167 beetle individuals and 66 morphospecies to emerge from the six-month-old bolts, 75 individuals and 27 morphospecies emerged from protected bolts. Almost all species collected from protected bolts are predators or fungus-feeders and some of these may have already been present when the bolts were originally enclosed. Interestingly, the most common species to emerge from protected bolts (23 individuals) was *Inopeplus reclusa* LeConte, a rarely-collected salpingid. To my knowledge, the mesh bags only failed to exclude a single species of phloem- or wood-consuming insect. Larvae and adults of the phloem-feeding cerambycid *Rhagium inquisitor* L. were observed under the bark of protected bolts after 6 and 24 months, respectively. This species may have successfully colonized protected bolts by ovipositing through the mesh screen. It should also be noted that although no failures were detected, the bags were beginning to show signs of deterioration by the end of the study (e.g., the wooden components were beginning to rot) and would probably have limited utility in longer term studies.

**Figure 6:**
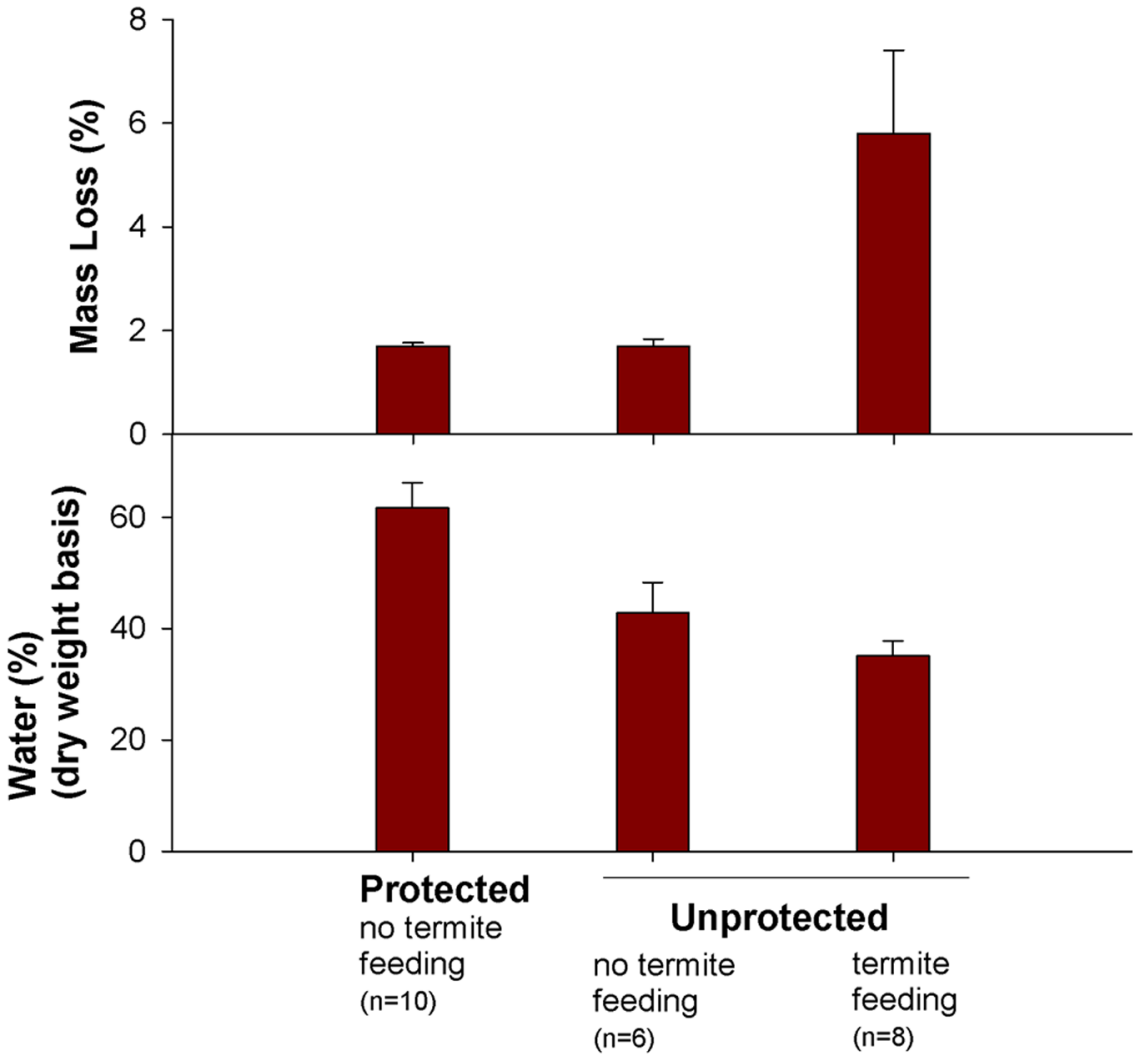
Mass loss and water content of protected and unprotected blocks after 20-weeks. Protected blocks were enclosed within stainless steel mesh for the duration of the experiment.

In the separate field study aimed at testing for unintended effects of the mesh bags beyond the exclusion of insects, water content varied significantly (Kruskal-Wallis H = 11.9, df = 2, P<0.01) among the three categories of blocks, being greatest for protected blocks (Figure 6). Mass loss varied significantly (H = 11.7, df = 2, P<0.01) among the three categories, being greatest for unprotected blocks with visible *Reticulitermes* damage (Figure 6). Most importantly, however, mass loss did not differ between protected blocks and unprotected blocks without visible *Reticulitermes* damage (Figure 6).

## Discussion

Because arthropods are larger, more mobile and, in certain cases (e.g., termites), capable of modifying the environment to meet their needs, they may not respond to physical conditions in the same direction or to the same extent as microbial decomposers. Termites have been shown to be relatively resilient to dry conditions, for instance, and are responsible for higher-than-expected decomposition rates in such environments [26], [44], [45]. By contrast, the findings from the current study suggest subterranean termite activity, like that of microbes, is negatively affected by periodic flooding. As predicted in hypothesis 1, seasonal flooding reduced *Reticulitermes* activity, with the effect being especially acute below-ground. These results are perhaps not surprising considering *Reticulitermes* builds diffuse subterranean nests and galleries [46]. Indeed, it is of interest that the insects were not even more strongly impacted given that the flooded sites were completely inundated for several weeks each year (Figure 2A). Whether these organisms were somehow able to survive locally or had to re-colonize these sites is not clear although several observations made by previous researchers suggest that *Reticulitermes* may find refuge from flooding in dead wood, including floating logs [34] and in standing dead trees [47].

Regardless of forest type, bolts unprotected from termites and other insects decomposed significantly faster than protected bolts, thus supporting hypothesis 2. Based on effect sizes and their 95% confidence intervals, insects significantly reduced specific gravity (initial volume) by 24 months in unflooded forests and by 31 months in flooded forests (Figure 5). While it is possible that the exclusion method used in the current study may also have affected decomposition beyond the exclusion of insects (e.g., by affecting wood moisture enough to influence microbial activity), two lines of evidence suggest this was not the case. First, only minor differences in wood moisture (i.e., the physical condition most likely to affect microbial activity) were observed between treatments over the first two years of the study and this was the case in both forest types (Figure 4). Second, the separate study aimed at testing for unintended effects of the mesh bags on wood decomposition found that even though water content was about 19% higher in protected blocks compared to unprotected blocks that were not attacked by insects, there was no difference in mass loss between the two treatments (Figure 6). Although the separate study was relatively short term and involved wooden blocks instead of the large bolts used in the main study, these findings lend additional credibility to the invertebrate effect reported herein. In a review of studies exploring the role of invertebrates in litter decomposition, Kampichler and Bruckner [37] recently stressed the need for such validation in exclusion studies. It is hoped that the novel method employed in the current study, or some similar approach, may have utility to future researchers interested in quantifying the contributions of invertebrates to wood decomposition.

Forest type had a significant effect on decomposition rates, with bolts placed in flooded forests decomposing more slowly than those placed in unflooded forests. Because this was the case for both protected and unprotected bolts and there was no significant treatment*forest interaction, this difference can be mostly attributed to the inhibition of microbial activity by seasonal flooding. By contrast, Hopkins [48] attributed differences in wood decomposition rates between a moist deciduous forest and a moist evergreen forest in Nigeria largely to termites. Wood decomposed much more rapidly in the former forest type with a termite incidence of 34% compared to the latter with a termite incidence of just 2%. Differences in above-ground termite incidence between bolts placed in flooded and unflooded forests in the current study were less extreme, ranging from 30–70% in flooded forests and from 50–100% in unflooded forests (Table 3). Because termites were 5–6 times more active below-ground in unflooded forests, however, seasonal flooding may substantially reduce the contributions of these organisms to subterranean wood decomposition. Because roots account for a considerable fraction of woody biomass in forests (approximately 20–25% of the above ground value [49]), research addressing this question would be of interest.

Ulyshen et al. [36] found specific gravity (final wood volume) to be significantly higher in bolts unprotected from termites, presumably because foraging termites leave behind the densest wood. Although no significant differences were detected between treatments in the current study, the same trend was evident in unflooded forests, with specific gravity (final wood volume) being consistently higher in unprotected bolts compared to protected bolts beginning at 12 months. The opposite pattern was consistently observed in flooded forests, however. These findings suggest the denser components of wood decompose more quickly in flooded forests than in unflooded forests, possibly due to differences in fungal community composition between forest types.

Providing some of the best evidence yet that arthropods play an important role in wood decomposition, the results from this study indicate that southeastern U.S. arthropod communities accelerate wood decomposition significantly and to a similar extent in both flooded and unflooded forests. Failure to recognize the contributions of these organisms to decomposition risks underestimating decomposition rates or exaggerating the importance of microbes to the process. Because above-ground termite activity was only weakly inhibited by seasonal flooding, however, the reduced decomposition rates observed in flooded forests compared to unflooded forests can be attributed largely to reduced microbial activity.
